# Extracellular Cyclic GMP Modulates Membrane Expression of The GluA1 and GluA2 Subunits of AMPA Receptor in Cerebellum: Molecular Mechanisms Involved

**DOI:** 10.1038/s41598-017-18024-3

**Published:** 2017-12-15

**Authors:** Andrea Cabrera-Pastor, Lucas Taoro-González, Amparo N. Cuñat, David Canet-López, Tiziano Balzano, Vicente Felipo

**Affiliations:** Laboratory of Neurobiology, Centro de Investigación Príncipe Felipe, 46012 Valencia, Spain

## Abstract

There is increasing evidence that extracellular cGMP modulates glutamatergic neurotransmission and some forms of learning. However, the underlying mechanisms remain unknown. We proposed the hypotheses that extracellular cGMP may regulate membrane expression of AMPA receptors. To do this extracellular cGMP should act on a membrane protein and activate signal transduction pathways modulating phosphorylation of the GluA1 and/or GluA2 subunits. It has been shown that extracellular cGMP modulates glycine receptors. The aims of this work were to assess: 1) whether extracellular cGMP modulates membrane expression of GluA1 and GluA2 subunits of AMPA receptors in cerebellum *in vivo*; 2) whether this is mediated by glycine receptors; 3) the role of GluA1 and GluA2 phosphorylation and 4) identify steps of the intracellular pathways involved. We show that extracellular cGMP modulates membrane expression of GluA1 and GluA2 in cerebellum *in vivo* and unveil the mechanisms involved. Extracellular cGMP reduced glycine receptor activation, modulating cAMP, protein kinases and phosphatases, and GluA1 and GluA2 phosphorylation, resulting in increased GluA1 and reduced GluA2 membrane expression. Extracellular cGMP therefore modulates membrane expression of AMPA receptors and glutamatergic neurotransmission. The steps identified may be therapeutic targets to improve neurotransmission and neurological function in pathological situations with abnormal glutamatergic neurotransmission.

## Introduction

AMPA receptors (AMPAR) are one type of glutamate receptors that mediate fast synaptic transmission in the central nervous system. They are tetrameric ligand-gated ion channels consisting of different combinations of four subunits (GluA1–4)^1^. The subunit composition determines synapse trafficking and functional properties of AMPAR^2–4^. For instance, the presence of the GluA2 subunit makes AMPAR calcium-impermeable^5^. In cerebellum, the fast excitatory transmission onto Purkinje neurons is mainly mediated by AMPAR^6^. Single-cell RT-PCR analysis revealed the presence of GluA1 and GluA2 in Purkinje neurons^7^.

One of the principle mechanisms regulating AMPAR function is the modulation of membrane expression of the subunits^8^. Increased and decreased membrane expression of AMPAR during long-term potentiation (LTP) and long-term depression (LTD) in the hippocampus and cerebellum, respectively, plays an essential role^4^. Homeostatic plasticity is also modulated via changing AMPAR trafficking processes^9^.

A key mechanism modulating membrane expression is phosphorylation^10^. Phosphorylation of GluA2 at Ser880 by protein kinase C (PKC) results in rapid internalization of GluA2-containing AMPA receptor^11–14^. Calcium/calmodulin-dependent kinase II (CaMKII) phosphorylates the GluA1 subunit at Ser831^15,16^, increasing AMPAR conductance and membrane expression^17,18^.

Modulation of membrane expression of AMPAR by modulating phosphorylation of GluA1 and GluA2 subunits is therefore an important mechanism in the modulation of glutamatergic neurotransmission and synaptic plasticity^2^. Different molecules and signaling cascades may modulate membrane expression of GluA1 and GluA2 through modulation of PKC and CaMKII^9^. For example, increases in calcium concentration activate adenylyl cyclases (AC) 1 and 8, which increase cAMP, activating cAMP-dependent protein kinase (PKA). PKA, in turn, phosphorylates inhibitor-1 (I-1) at Thr35 which reduces protein phosphatase 1 (PP1) increasing CaMKII phosphorylation and activity^19^. These pathways may be induced by different physiological or pathological molecules such as TNF-a^9^ or NMDA receptor activation by glutamate^19^.

The roles of cGMP as an intracellular second messenger are well known. There is increasing evidence that extracellular cGMP, at physiological concentrations, also exerts an important function in modulating cerebral activity. Physiological roles for extracellular cGMP have been reported in kidney Na+ transport^20^ and in intestinal motility^21^. Extracellular cGMP also modulates some aspects of cerebral function. In primary cultures of astrocytes extracellular cGMP modulates a Na^+^/H^+^ exchanger^22^ and in isolated rat hippocampal pyramidal neurons modulates glycine receptors^23^. *In vivo* administration of extracellular cGMP also modulates membrane expression of AMPA receptors in hippocampus^24^.

Extracellular cGMP seems to play a relevant role especially in cerebellum. Linden *et al*.^25^ reported that extracellular cGMP reduces the glutamate-evoked currents involved in long-term depression in Purkinje neurons. Cervetto *et al*.^26^ reported that extracellular cGMP inhibits glutamate release evoked by presynaptic kainate receptors in cerebellar parallel/climbing fibers. These two studies were performed using cGMP concentrations (from micro- to milli-molar) much higher than physiological extracellular cGMP levels (nanomolar) in cerebellum. However, other reports have also shown effects of extracellular cGMP in cerebellum at physiological concentration. Montoliu *et al*.^27^ showed that extracellular cGMP at physiological (nanomolar) concentration prevents glutamate-induced death in primary cultures of cerebellar neurons. Extracellular cGMP at nanomolar concentrations also reduces glycine receptors activation in Purkinje neurons, thus modulating intracellular calcium and CaMKII activity^28^. Extracellular cGMP also modulates the ability to learn a Y-maze task in rats *in vivo*. Altered extracellular cGMP levels in cerebellum may induce cognitive impairment. For example, rats with chronic hyperammonemia show reduced extracellular cGMP levels ion cerebellum and impaired ability to learn this task. Chronic intracerebral administration of extracellular cGMP normalized extracellular cGMP in cerebellum of hyperammonemic rats and restored their ability to learn the Y maze task^28^. These few reports support the idea that extracellular cGMP, at physiological concentrations, modulates different aspects of the cerebellum function *in vivo*, as well as some forms of learning (Y maze task) modulated by the cerebellum. However, the underlying mechanisms remain largely unknown. Altogether, these studies suggest that extracellular cGMP seem to play relevant physiological roles in brain and, especially in cerebellum. We therefore decided to focus the present work in cerebellum.

Based on the above data we suggest the hypotheses that extracellular cGMP may regulate membrane expression of AMPA receptor in cerebellum by modulating glycine receptors and signal transduction pathways leading to changes in GluA1 and GluA2 phosphorylation.

The aims of this work were to: 1) assess whether chronic administration of extracellular cGMP modulates membrane expression of GluA1 and GluA2 subunits of AMPA receptors in cerebellum; 2) assess whether this is mediated by modulation of glycine receptors; 3) assess the role of GluA1 and GluA2 phosphorylation and 4) identify steps of the intracellular pathways involved.

## Results and Discussion

### Intracerebral administration of extracellular cGMP modulates in opposite ways the membrane expression of GluA1 and GluA2 subunits of AMPA receptor in cerebellum ***in vivo***

**Figure 1:**
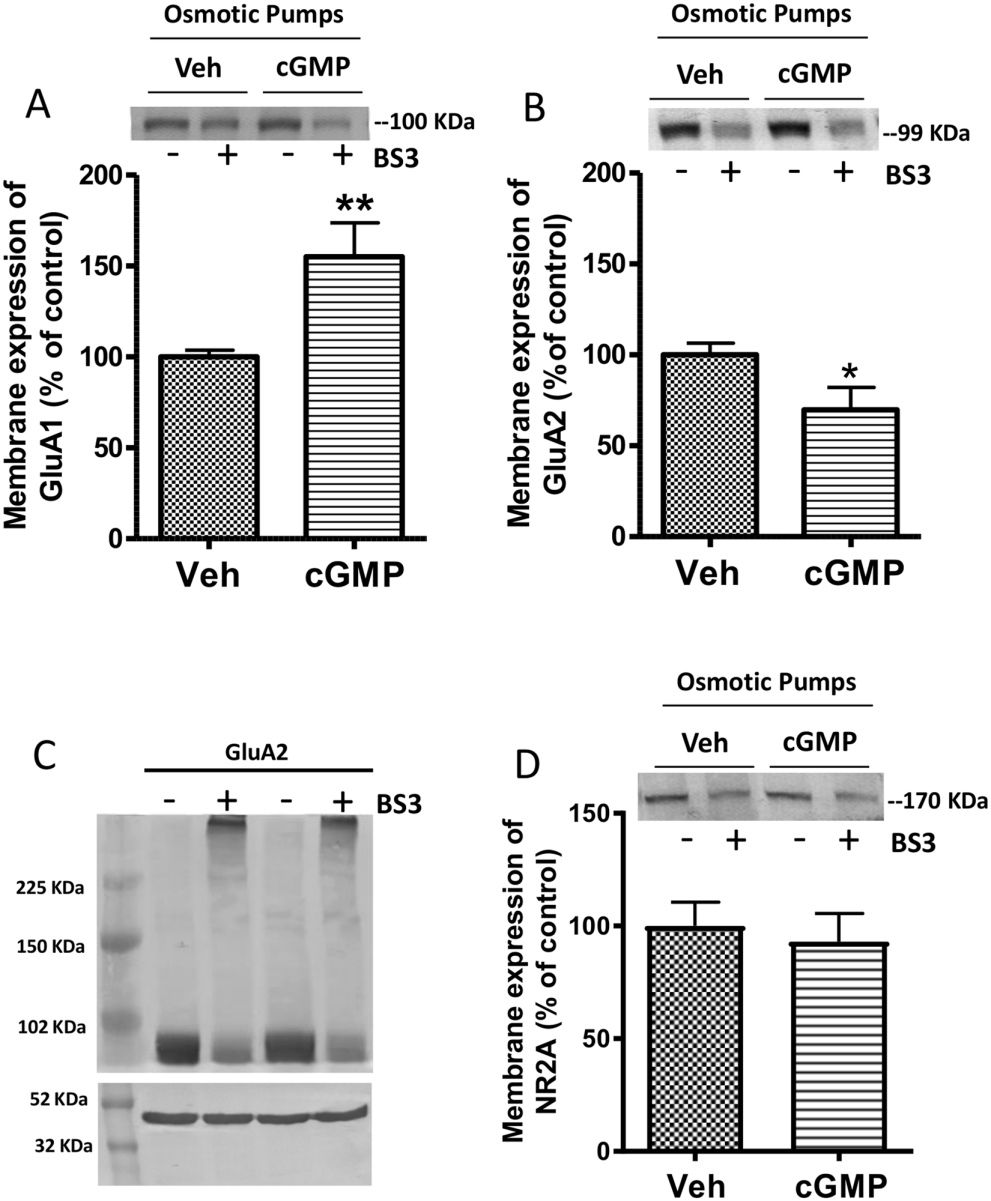
Chronic intracerebral administration of extracellular cGMP increases membrane expression of the GluA1 (**A**) and decreases GluA2 (**B**) subunits of AMPA receptors in cerebellum *in vivo*. Membrane expression of NR2A in the same samples is not affected by extracellular cGMP (**D**). For one group of rats the osmotic pumps were filled with 240 µM cGMP in sterile saline (cGMP) and for the other group with the vehicle solution, sterile saline (veh). These pumps released 0.25 μL per hour during 28 days. Cerebellum of rats was dissected and membrane expression of each subunit was analyzed using the BS3 crosslinker procedure as described in Methods. (**C**) Monomeric and cross-linked bands of GluA2 and actin from western blot are shown. Values are expressed as percentage of control rats (vehicle) and are the mean ± SEM of 16–19 rats per group. The unpaired Student’s t-test was performed. Values significantly different are indicated by asterisks *p < 0.05, **p < 0.01.

We analyzed the effects of continuous intracerebral administration of cGMP, which does not cross the cell membrane^22^, on membrane expression of GluA1 and GluA2 in cerebellum. Chronic administration of extracellular cGMP induced opposite effects on membrane expression of the subunits of AMPA receptor, increasing GluA1 to 155 ± 18% (p < 0.01) of basal (Fig. 1A) and decreasing (p < 0.05) GluA2 to 69 ± 12% (Fig. 1B and C). This was a selective effect and not a general effect of extracellular cGMP on all membrane proteins. The membrane expression of NR2A in the same samples was not affected by extracellular cGMP (Fig. 1D and Supplementary Fig. 1).

### Addition of extracellular cGMP to slices of cerebellum reproduces the effects on membrane expression of GluA1 and GluA2 subunits of AMPA receptor *in vivo*

**Figure 2:**
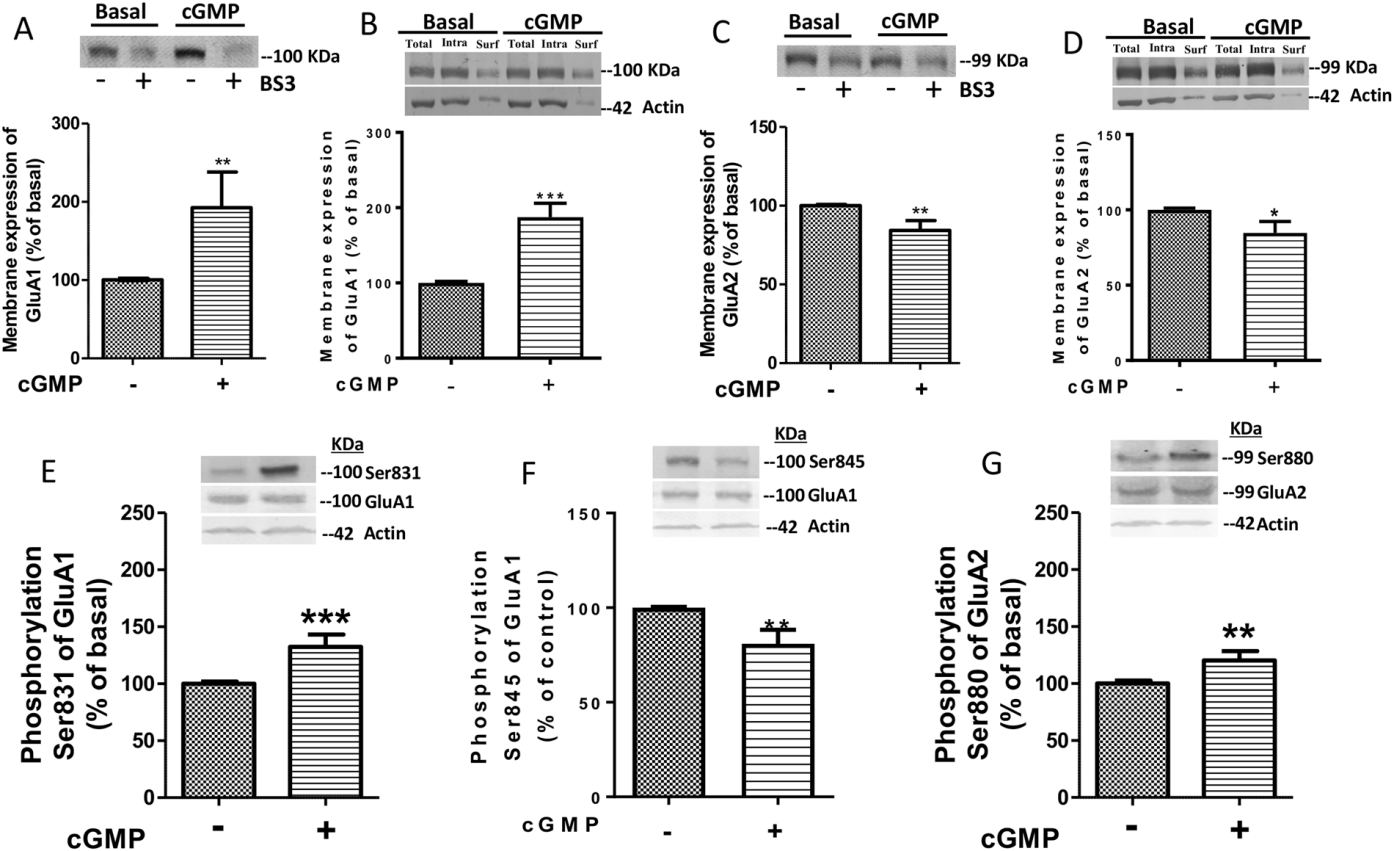
Addition of extracellular cGMP to cerebellar slices modulates membrane expression of AMPA receptor subunits and phosphorylation of Ser831 of GluA1 and Ser880 of GluA2. Exogenous cGMP (40 nM) was added to freshly isolated cerebellar slices. Membrane expression of GluA1 (**A** and **B**) and GluA2 (**C** and **D**) subunits were analyzed by BS3 crosslinking method (**A** and **C**) and by biotinylation assay (**B** and **D**) and their phosphorylation at Ser831 (**E**), Ser845 (**F**) and Ser880 (**G**), respectively, were analyzed as described in Methods. Values are expressed as percentage of basal and are the mean ± SEM of 20–30 rats. The unpaired Student’s t-test was performed. Values significantly different are indicated by asterisks *p < 0.05, **p < 0.01, ***p < 0.001.

The above results show that extracellular cGMP modulates membrane expression of AMPA receptor subunits in cerebellum *in vivo*. As the molecular mechanisms involved could not be analyzed in full detail *in vivo*, we assessed whether addition of exogenous cGMP to freshly isolated cerebellar slices reproduced the effects on membrane expression of GluA1 and GluA2. Addition of cGMP (40 nM) increased membrane expression of GluA1 to 185 ± 43% (p < 0.01) of basal (Fig. 2A) and reduced GluA2 to 84 ± 6% (p < 0.01) (Fig. 2C), as analyzed using the BS3 procedure, thus reproducing the effects found *in vivo*. These results were confirmed by analyzing surface expression of GluA1 and GluA2 by the biotinylation procedure. Addition of cGMP (40 nM) increased membrane expression of GluA1 to 188 ± 18% (p < 0.001) of basal (Fig. 2B) and reduced GluA2 to 85 ± 7% (p < 0.05) (Fig. 2D), as analyzed using the biotinylation procedure, thus reproducing the effects found *in vivo*. Representative images of the biotinylation analysis are shown in Supplementary Fig. 2).

Freshly isolated cerebellar slices are therefore a good system to study the mechanisms by which extracellular cGMP modulates membrane expression of the AMPA receptor subunits.

### Mechanisms involved in the regulation of GluA1/A2 phosphorylation and membrane expression by extracellular cGMP

As indicated in the introduction, membrane expression of GluA1 and GluA2 are modulated by phosphorylation of Ser831 and Ser880, respectively. We therefore analyzed the effects of cGMP (40 nM) on the phosphorylation of these residues in cerebellar slices.

Extracellular cGMP increased phosphorylation of Ser831 of GluA1 to 133 ± 11% (p < 0.001) of basal (Fig. 2E) and of Ser880 of GluA2 to 120 ± 8% (p < 0.01) (Fig. 2G).

Extracellular cGMP also decreased Ser845phosphorylation of GluA1 to 81 ± 7% (p < 0.01) (Fig. 2F). This result indicated that extracellular cGMP modulated phosphorylation of GluA1 at Ser831 and of GluA2 at Ser880, which could mediate the effects on membrane expression of these subunits.

**Figure 3:**
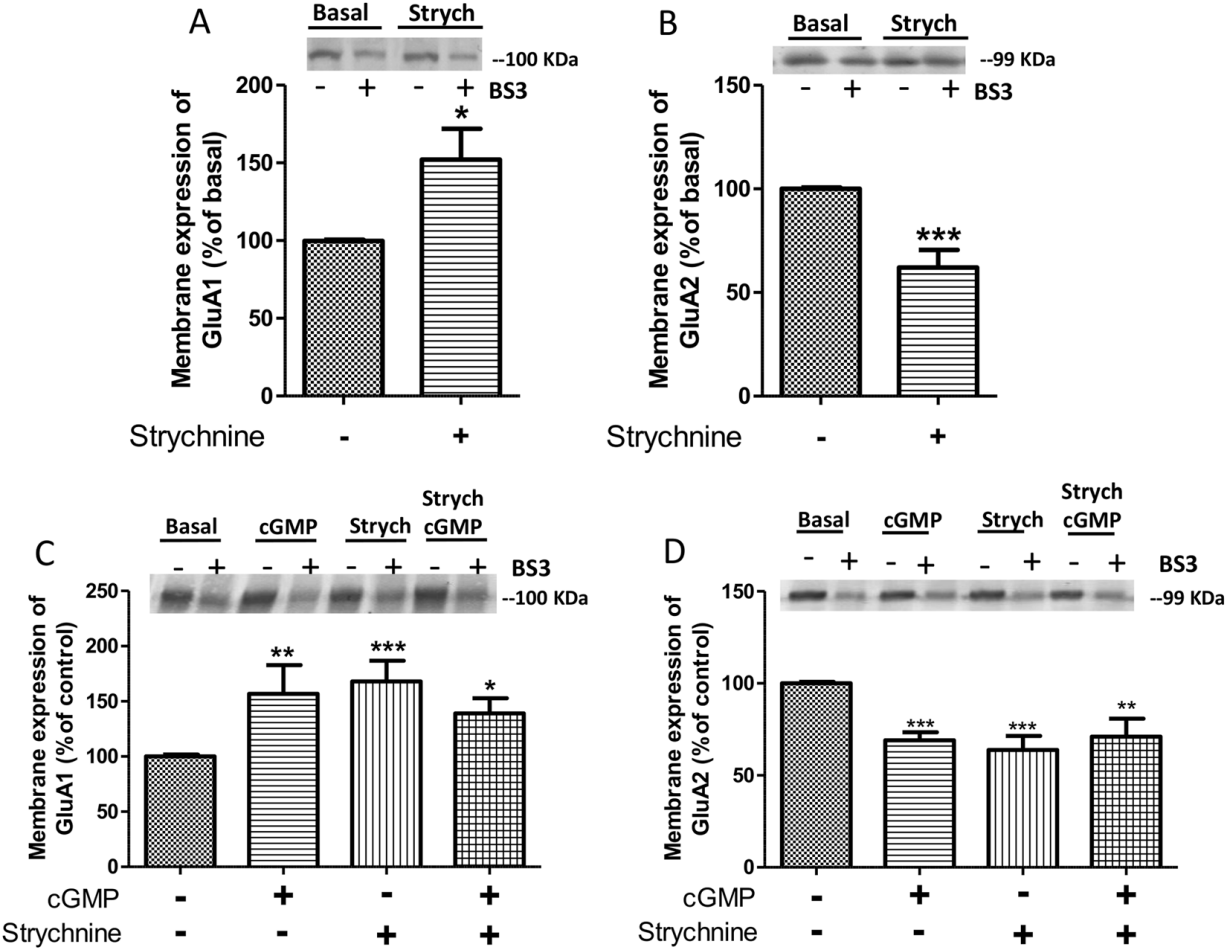
Strychnine, an antagonist of the glycine receptor induces the same effects as extracellular cGMP on membrane expression and occludes the effect of cGMP on membrane expression of GluA1 and GluA2. Strychnine (75 µM), an antagonist of glycine receptor, alone or in combination with extracellular cGMP (40 nM) were added to cerebellar slices, and membrane expression of GluA1 (**A** and **C**) and GluA2 (**B** and **D**) subunits were analyzed as described in Methods. Values are expressed as percentage of basal and are the mean ± SEM of 8–16 rats (in **A** and **B**) and of 7–12 rats (in **C** and **D**). The unpaired Student’s t-test and non-parametric Kruskal-Wallis test were performed to compare two or more groups, respectively. Values significantly different from basal are indicated by asterisks *p < 0.05, **p < 0.01, ***p < 0.001.

To modulate intracellular phosphorylation of AMPA receptor subunits, extracellular cGMP must act on some membrane protein, leading to activation of signal transduction pathways that modulate protein kinases and/or phosphatases modulating AMPA receptor subunits phosphorylation. Extracellular cGMP has been shown to modulate the glycine receptor^23^. To assess if modulation of membrane expression of AMPA receptor subunits by extracellular cGMP is mediated by glycine receptors we assessed if an antagonist, strychnine, induces similar effects. Strychnine did not affect the total amount of GluA1 and GluA2, which remained at 104 ± 6% and 91 ± 11% of basal, respectively. As shown in Fig. 3, strychnine induced the same effects as extracellular cGMP, increasing membrane expression of GluA1 to 152 ± 19% (p < 0.05) (Fig. 3A) and reducing that of GluA2 to 62 ± 8% (p < 0.001) (Fig. 3B).

These results suggest that modulation of membrane expression of AMPA receptor subunits by extracellular cGMP is mediated by reduction of glycine receptor activation. To confirm this possibility we performed experiments to assess whether strychnine pharmacologically occludes the effect of cGMP on membrane expression of AMPA receptor subunits. If cGMP induces the effects on membrane expression of AMPAR subunits by reducing the activation of the glycine receptor, its effects should be prevented by blocking the receptor with strychnine. If the glycine receptor is already blocked by strychnine, adding cGMP should not block it further and therefore the effects of strychnine +cGMP would be the same as those of strychnine or cGMP alone.

However, if cGMP induced the effects on membrane expression by a different mechanism, independent of glycine receptor, strychnine should not prevent the effects of cGMP. The mixture of cGMP + strychnine should therefore induce additive effects on the changes in membrane expression. The combination of strychnine and extracellular cGMP induced the same effects as the individual administration of each compound alone on membrane expression of GluA1 and GluA2 subunits. In these experiments cGMP alone increased membrane expression of GluA1 to 157 ± 26% of basal (p < 0.01), strychnine alone to 168 ± 19% (p < 0.001) and the combination of cGMP + strychnine to 140 ± 14% (p < 0.05), (Fig. 3C). Concerning GluA2, cGMP alone reduced membrane expression of GluA2 to 69 ± 4% of basal (p < 0.001), strychnine alone to 64 ± 8% (p < 0.001) and the combination of cGMP + strychnine to 71 ± 10% (p < 0.05) of basal (Fig. 3D). This confirmed the hypothesis that blocking glycine receptors with strychnine occluded the effect of cGMP, supporting that cGMP modulates membrane expression of AMPA receptor subunits by reducing glycine receptor activation.

**Figure 4:**
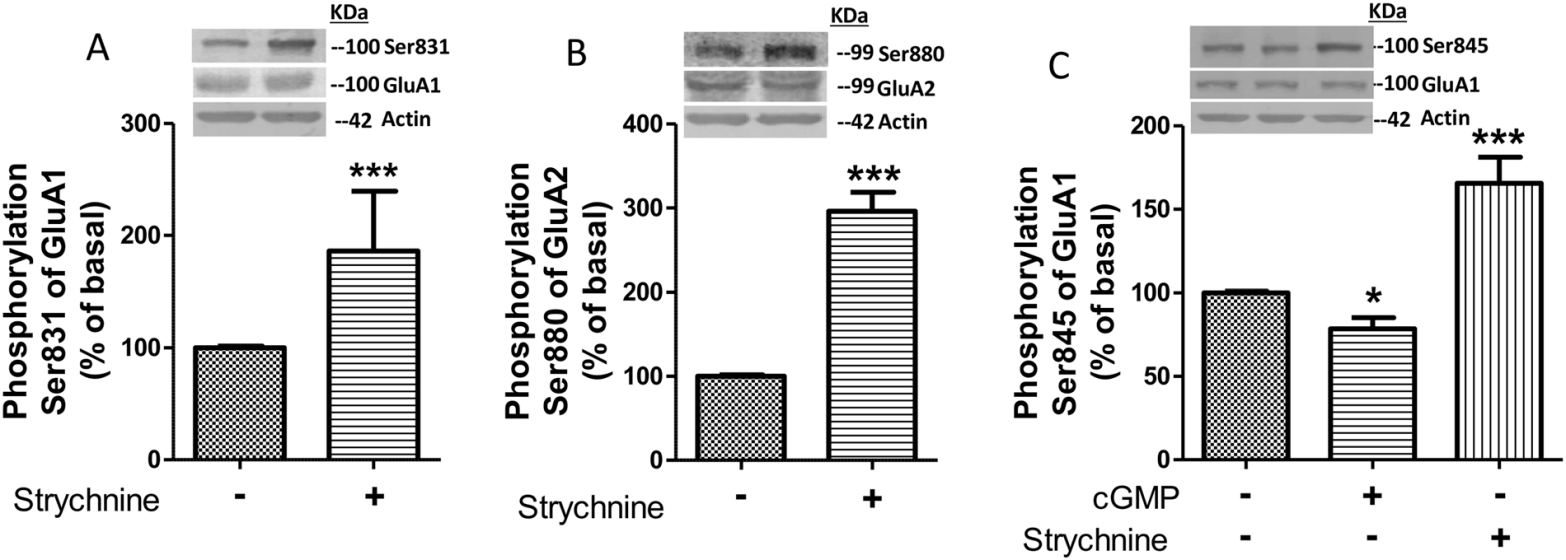
Blocking glycine receptors with strychnine induces the same the effects as extracellular cGMP on phosphorylation at Ser831 of GluA1 and at Ser880 of GluA2 subunits but not at Ser845 of GluA1. Strychnine (75 µM), an antagonist of glycine receptor, was added to cerebellar slices and phosphorylation at Ser831 (**A**), Ser880 (**B**) and Ser845 (**C**) were analyzed as described in Methods. Values are expressed as percentage of basal and are the mean ± SEM of 8–16 rats. The unpaired Student’s t-test was performed, Values significantly different from basal are indicated by asterisks *p < 0.05, ***p < 0.001.

Strychnine also affected phosphorylation of GluA1 at Ser831 and of GluA2 at Ser880 similarly to extracellular cGMP, increased phosphorylation of Ser831 of GluA1 to 186 ± 53% (p < 0.001) of basal (Fig. 4A) and that of Ser880 of GluA2 to 296 ± 22% (p < 0.001) of basal (Fig. 4B). However, strychnine affects phosphorylation of GluA1 at Ser845 differently than extracellular cGMP, increasing it to 166 ± 16% (p < 0.001) of basal (Fig. 4C).

This suggests that the effects of extracellular cGMP on phosphorylation of GluA1 at Ser831 and of GluA2 at Ser880 would be mediated by the glycine receptor while the effects on phosphorylation of GluA1 at Ser845 are mediated by a different mechanism. As the effects of extracellular cGMP and that of strychnine on membrane expression are similar, this suggests that phosphorylation of GluA1 at Ser845 would not play a main role in the effects of extracellular cGMP on membrane expression and Ser831 of GluA1 and Ser880 of GluA2 would be more relevant.

Ser831 of GluA1 and Ser880 of GluA2 are phosphorylated by PKC and CaMKII^9^. We then assessed the effects of inhibiting these protein kinases on phosphorylation and membrane expression of the AMPA receptor subunits.

**Figure 5:**
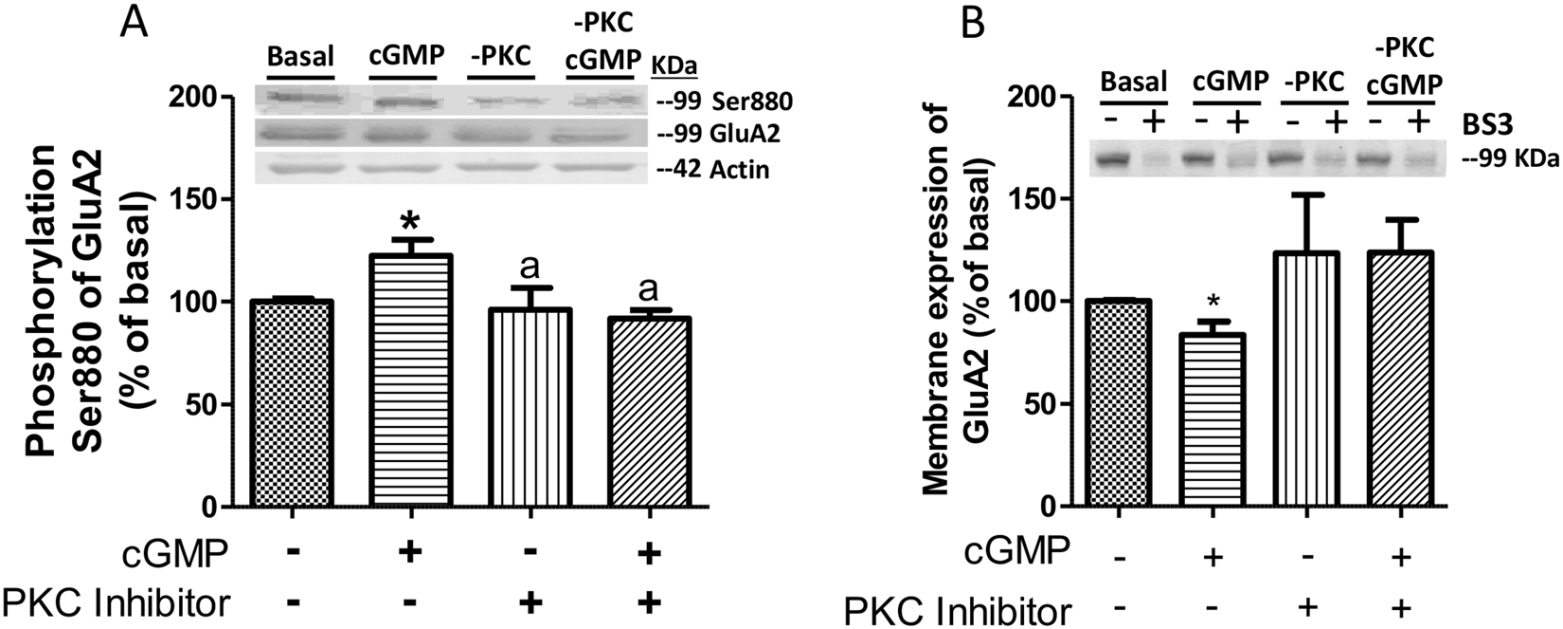
Reduction of GluA2 membrane expression induced by extracellular cGMP is mediated by activation of PKC. Extracellular cGMP was added to cerebellar slices in the absence or the presence of an inhibitor of PKC (1 µM Bisindolylmaleimide II). Phosphorylation at Ser880 (**A**) and surface expression of GluA2 subunit (**B**) were analyzed as described in Methods. Values are expressed as percentage of basal and are the mean ± SEM of 8 rats. Data were analyzed by one-way analysis of variance (in **A**) and by the non-parametric Kruskal-Wallis test (in **B**).Values significantly different from basal are indicated by asterisk *p < 0.05. Values significantly different from cGMP treatment are indicated by a p < 0.05.

Inhibition of PKC prevented the effects of extracellular cGMP on phosphorylation of Ser880 of GluA2, which remained at 91 ± 5% (Fig. 5A) and on membrane expression of GluA2, which remained at 115 ± 15% (Fig. 5B). It has been shown that phosphorylation of GluA2 at Ser880 by protein kinase C (PKC) results in rapid internalization of GluA2-containing AMPA receptors^11–14^. This suggests that the effects of extracellular cGMP on membrane expression of GluA2 could be mediated by a PKC.

**Figure 6:**
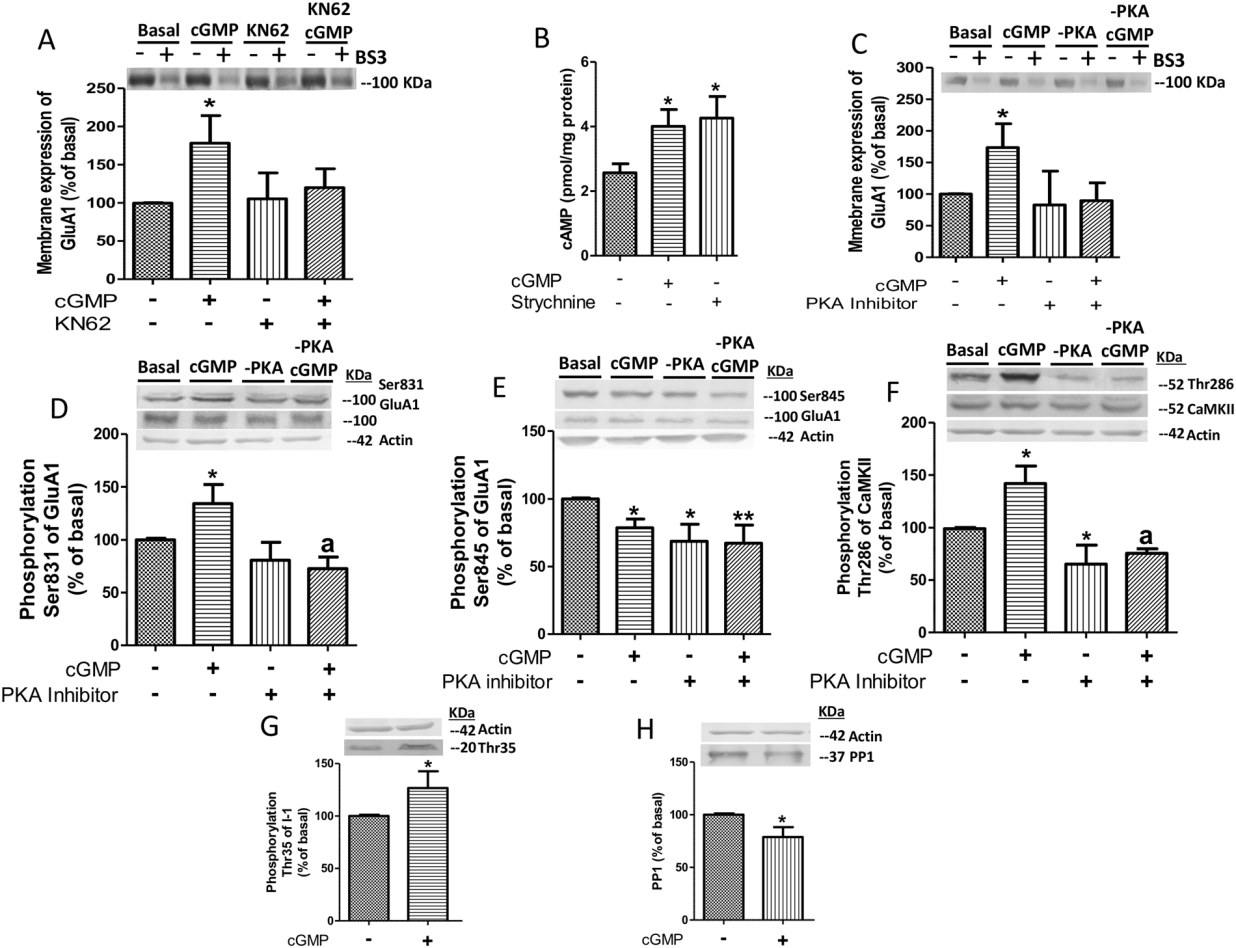
Extracellular cGMP increases membrane expression of GluA1 subunit through activation of the cAMP-PKA-I1-PP1-CaMKII pathway. (**A**) Extracellular cGMP was added to cerebellar slices in the absence or the presence of an inhibitor of CaMKII (KN62, 10 µM) and membrane expression of GluA1 was analyzed. (**B**) cAMP levels in cerebellar slices after administration of 40 nM cGMP or 75 µM strychnine. (**C**–**F**) Extracellular cGMP was added to cerebellar slices in the absence or the presence of an inhibitor of PKA (1 µM 14–22 Amide) and membrane expression of GluA1 (**C**) and its phosphorylation at Ser831 (**D**), at Ser845 (**E**) and phosphorylation of CaMKKI at Thr286 (**F**) were analyzed. (**G**,**H**) Content of protein phosphatase inhibitor-1 (I1) phosphorylated at Thr 35 (**G**) and PP1 (**H**) after treatment with extracellular cGMP. Non-parametric Kruskal-Wallis test (in **A**,**C**–**F**), one-way analysis of variance (in **B**) and the unpaired Student’s t-test (in **G,H**) were performed. Values are expressed as percentage of basal and are the mean ± SEM of 10–15 rats. Values significantly different from basal are indicated by asterisk *p < 0.05, **p < 0.01. Values significantly different from cGMP treatment are indicated by a p < 0.05.

Inhibition of CaMKII prevented the effects of extracellular cGMP on membrane expression of GluA1, which remained at 119 ± 24% of basal (Fig. 6A).

This suggested that the effects of extracellular cGMP on membrane expression of GluA1 are mediated by CaMKII.

CaMKII is activated by calcium-calmodulin^29^, and once activated, it is autophosphorylated at Thr286^30^. Once phosphorylated at this site, CaMKII is autonomously active^31^. Phosphorylation of CaMKII at Thr286 is therefore an indicator of CaMKII activity.

CaMKII may be activated directly by the increase in intracellular calcium induced by extracellular cGMP but also through the cAMP-PKA-I1P (Thr35)-PP1 pathway described in the introduction. We assessed the possible role of this pathway.

We first analyzed the effects of reducing glycine receptors activation on intracellular cAMP. Both extracellular cGMP and strychnine increased intracellular cAMP levels, reaching 4 ± 0.5 and 4.2 ± 0.6 pmol/mg protein, respectively (Fig. 6B). These values are significantly higher (p ˂ 0.05) than basal values (2.6 ± 0.3 pmol/mg protein).

We then analyzed the effects of inhibiting PKA. Inhibition of PKA prevented the effects of extracellular cGMP on membrane expression of GluA1 subunit, which remained at 89 ± 28% of basal (Fig. 6C) and on its phosphorylation at Ser831, which remained at 73 ± 11% of basal (Fig. 6D). Inhibition of PKA did not affect phosphorylation of GluA1 at Ser845 (Fig. 6E).

Inhibition of PKA also prevented activation of CaMKII by extracellular cGMP. Activation of CaMKII is reflected in increased phosphorylation of Thr286 (see above). As shown in Fig. 6F, extracellular cGMP increased phosphorylation of Thr286 (reflecting increased activity) of CaMKII, reaching 142 ± 16% of basal (p < 0.05). Inhibition of PKA completely prevented activation of CaMKII by extracellular cGMP, reducing phosphorylation of Thr286 to 75 ± 4% of basal (p < 0.001), which is similar to the phosphorylation of control slices treated with the PKA inhibitor (Fig. 6E). These data suggest that PKA mediates the effects of extracellular cGMP on CaMKII and on membrane expression of GluA1. We then assessed how PKA modulates CaMKII. PKA, through phosphorylation of the inhibitor I-1, modulates protein phosphatase PP1 which dephosphorylates and reduces the activity of CaMKII^32^.

Addition of extracellular cGMP activates PKA and increases phosphorylation of I-1 at Thr35 to 126 ± 16% (p < 0.05) (Fig. 6G) and reduced PP1 to 78 ± 9% (p <  0.05) (Fig. 6H) which is associated with increased phosphorylation of CaMKII at Thr286 (Fig. 6F).

**Figure 7:**
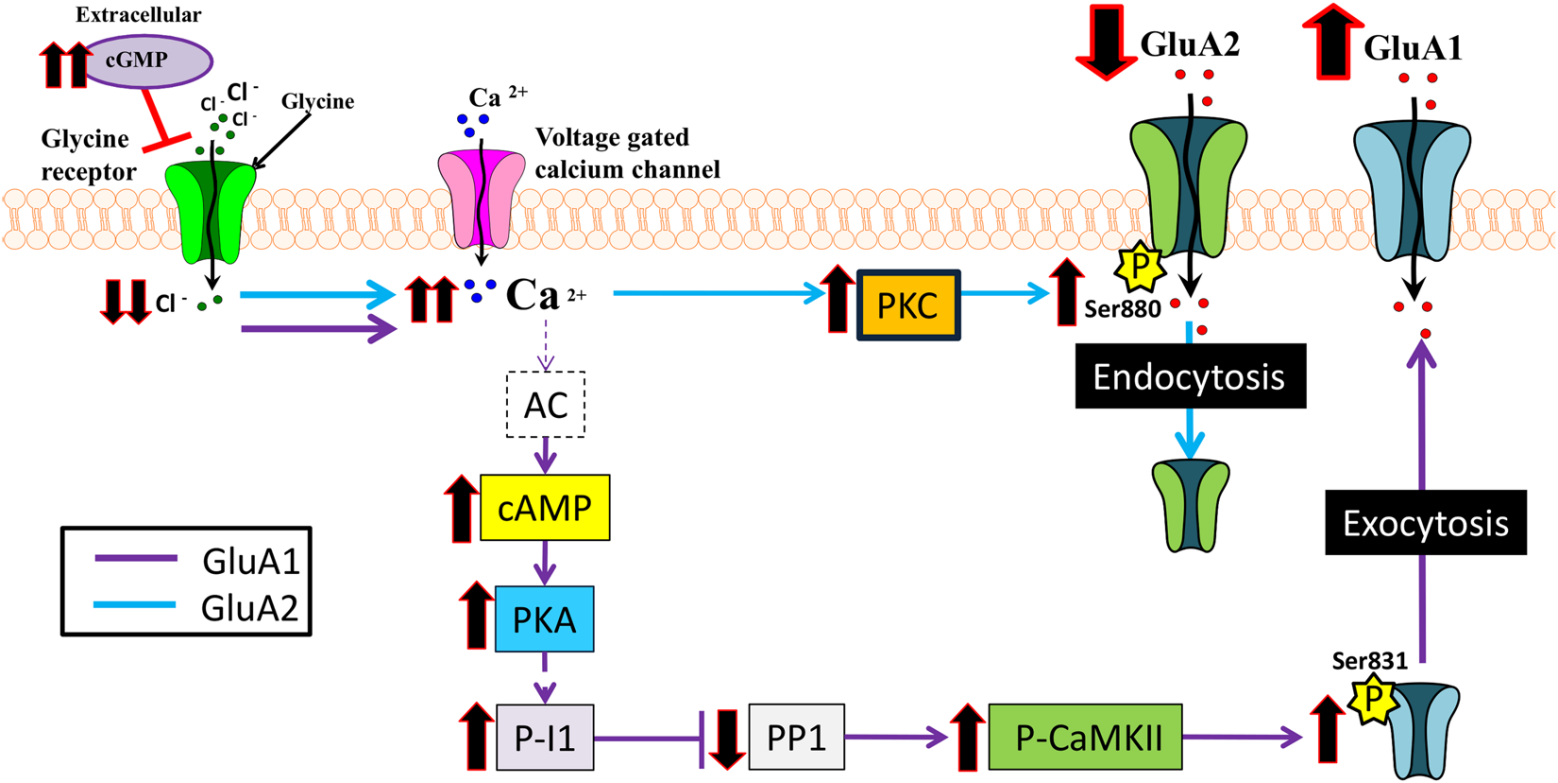
Model proposed about some mechanisms by which extracellular cGMP would modulate membrane trafficking of GluA1 and GluA2 subunits of AMPA receptor in cerebellum. Extracellular cGMP inhibits glycine receptors. This increases Ca^2+^ and activates PKC which increases phosphorylation of GluA2 at Ser880, thus reducing its membrane expression. Increased membrane expression of GluA1 is due to its phosphorylation at Ser831 which is mediated by activation of the cAMP-PKA-I1-PP1-CaMKII pathway by extracellular cGMP. The effects induced by extracellular cGMP on each parameter are indicated by the black arrows.

These results suggested that the effects of extracellular cGMP on membrane expression of GluA1 are mediated by the cAMP-PKA-I1-PP1 pathway, which activates the CaMKII, increasing its phosphorylation at Thr286, which would phosphorylate GluA1 at Ser831, increasing GluA1 membrane expression (Fig. 7). The increased activity of CaMKII could also explain the reduction of phosphorylation of GluA1 at Ser845. It has been reported that, in hippocampus, AKAP79 localizes PKA to the GluA1 C-terminus via SAP97 to allow phosphorylation of Ser845 by PKA^33^. When CaMKII is activated by autophosphorylation, it binds and phosphorylates SAP97, disrupting the GluA1-AKAP79-PKA complex^33^, which would reduce phosphorylation of Ser845 by PKA. However, it has been reported that the expression of AKAP79 is low in cerebellum^34^, making unlikely that exactly this mechanism would be present. It is possible that in cerebellum an alternative AKAP could play a similar role to AKAP79 in hippocampus or that CaMKII may modulate phosphorylation of GluA1 at Ser845 by an alternative mechanism.

As discussed below and summarized in Fig. 7, the data reported suggest that extracellular cGMP, through modulation of glycine receptor increased intracellular Ca^2+^ influx leading to the activation of adenylyl cyclase, PKA, PKC and CaMKII, which contribute to modulate phosphorylation and membrane expression of GluA1 and GluA2. It should be acknowledged that the signaling pathways activated by extracellular cGMP are probably even more pleiotropic than presented in Fig. 7. The increase in calcium induced by cGMP would modulate a myriad of signaling pathways, many of which could modulate phosphorylation of GluA1 and GluA2. The cartoon in Fig. 7 includes the main components of the response to extracellular cGMP which would contribute to modulation of phosphorylation and membrane expression of GluA1 and GluA2, but this does not exclude the possibilities that other pathways could be also affected by extracellular cGMP.

Taking this into account, we also assessed whether cGMP-dependent protein kinase (PKG) could also contribute to extracellular cGMP-mediated changes in GluA2 and GluA1 phosphorylation and membrane expression. Inhibition of PKG per se, in the absence of added extracellular cGMP, increased membrane expression of GluA2 (Supplementary Fig. 3A), indicating that PKG may modulate membrane expression of GluA2 under basal conditions. However, this is not associated with changes in phosphorylation of GluA2 at Ser880 (Supplementary Fig. 3B). Inhibition of PKG did not affect changes in phosphorylation (Supplementary Fig. 3B) or membrane expression (Supplementary Fig. 3A) of GluA2 induced by extracellular cGMP. These data suggests that, although PKG may modulate membrane expression of GluA2 under basal conditions, this kinase does not seem to play a relevant role in the effects of extracellular cGMP on GluA2.

Inhibition of PKG did not affect phosphorylation of GluA1 at Ser831 (Supplementary Fig. 3C) but reduced its phosphorylation at Ser845 (Supplementary Fig. 3D) and had a tendency to reduce membrane expression of GluA1 (Supplementary Fig. 3E), likely due to reduced phosphorylation of Ser845. Inhibition of also PKG did not prevent the effects of extracellular cGMP on phosphorylation of GluA1 at Ser831 (Supplementary Fig. 3C). However, inhibition of PKG in the presence of extracellular cGMP strongly reduced phosphorylation of GluA1 at Ser845 (Supplementary Fig. 3D) and prevented the increase in membrane expression of GluA1 induced by extracellular cGMP (Supplementary Fig. 3E). This indicates that inhibition of PKG does not affect the pathway depicted in Fig. 7, but may affect membrane expression of GluA1 by modulating phosphorylation at Ser845 through a different pathway (as mentioned above), but we do not exclude the possibilities that other pathways could also be affected by extracellular cGMP.

It is of note that activation of intracellular pathways by extracellular cGMP (shown in Fig. 7) might also lead to adaptive feedback responses. For example, activation of PKA could lead to later feedback modulation of glycine receptors. Activation of PKA could also increase phosphorylation of the alpha subunit of glycine receptors, which results in enhanced of glycine-evoked currents in Xenopus oocytes^35^. PKA activation also enhances the affinity of the receptors for glycine in ventral tegmental area neurons^36^. In contrast, in retina, PKA activation leads to an internalization of glycine receptors, reducing its amount in the cell surface and the binding of glycine^37^. It is therefore possible that activation of PKA in response to extracellular cGMP-induced reduction of glycine receptor activation could lead to a feedback modulation of glycine receptors in cerebellum.

The signaling pathways involving calcium, PKA, and the other components indicated in Fig. 7 are clearly pleiotropic and more pathways should be activated and feedback mechanisms may be triggered later, however, the data reported support that the steps shown in Fig. 7 are involved in the modulation of GluA1 and GluA2 membrane expression by extracellular cGMP.

The data reported here identify one mechanism by which extracellular cGMP modulates glutamatergic neurotransmission, which may modulate cognitive function. The results reported show that extracellular cGMP modulates membrane expression of GluA1 and GluA2 subunits of AMPA receptors in cerebellum *in vivo*. Moreover, the effects of extracellular cGMP on each subunit were the opposite; it increases membrane expression of GluA1 and reduces that of GluA2. These effects were reliably reproduced in freshly isolated cerebellar slices by addition of 40 nM cGMP or strychnine, an antagonist of glycine receptors. This supported our hypothesis that reducing upstream activation of glycine receptor mediated the effects of extracellular cGMP on GluA1 and GluA2 membrane expression. Using the cerebellar slice system we have identified key elements of the intracellular pathways by which extracellular cGMP modulates membrane expression of GluA1 and GluA2, which are summarized in Fig. 7.

Extracellular cGMP reduces activation of glycine receptors^23^. Bukanova *et al*.^23^ have shown that in rat hippocampal pyramidal neurons, extracellular cGMP modulates glycine-activated currents by accelerating the glycine-evoked chloride current desensitization in a voltage-independent and non-competitive manner. The effect was equally pronounced at negative and positive membrane potentials and already occurs at nanomolar (physiological) extracellular cGMP concentrations, being maximum at around 100 nM cGMP.

We have previously shown, in rat cerebellar slices, that modulation of glycine receptors by extracellular cGMP resulted in reduced intracellular Cl^−^ concentration and increased intracellular Ca^2+^ ^28^. Increased calcium activates PKC, which phosphorylates GluA2 at Ser880. It has been shown that this phosphorylation leads to internalization of GluA2-containig AMPARs^11–14^. Extracellular cGMP is therefore likely to reduce GluA2 membrane expression by increasing its phosphorylation at Ser880 by PKC (Fig. 7).

Concerning GluA1, extracellular cGMP increases phosphorylation of Ser831. This phosphorylation leads to increased membrane expression^17,18^. The mechanism by which extracellular cGMP increases phosphorylation of GluA1 involved reduction of glycine receptor activation, leading to increased cAMP, which activates PKA, increasing phosphorylation of I-1 at Thr35, reducing protein phosphatase PP1 which leads to increased phosphorylation and activity of CaMKII and phosphorylation of GluA1 at Ser831 (Fig. 7). The involvement of this pathway is supported by the fact that inhibiting PKA prevented all subsequent steps: activation of CaMKII, phosphorylation of GluA1 at Ser831 and increased membrane expression of GluA1.

Similar modulation of CaMKII activity by PKA through modulation of PP1 also occurs in the modulation of rebound potentiation (RP) at inhibitory synapses on Purkinje cells^38,39^. In this case PP1 is not modulated by I-1 but by a similar inhibitor, DARP32. To suppress RP, activation of GABAB receptors leads to down-regulation of PKA activity, resulting in reduced phosphorylation of DARP32, increased PP1 activity, reduced CaMKII phosphorylation (and activity) which alters phosphorylation of GABARAP and trafficking of GABAA receptors to the membrane^39^. It is therefore possible that, in addition to AMPA receptor-mediated neurotransmission, extracellular cGMP could also modulate GABAergic neurotransmission in cerebellum.

In cerebellum, AMPA receptors are mainly located in Purkinje cells, both at synapsis with parallel fibers and with climbing fibers^40^. To assess if extracellular cGMP could act on the glycine receptor and affect AMPAR in Purkinje neurons, we analyzed by immunohistochemistry the expression of glycine receptor and of GluA1 and GluA2 subunits in cerebellum. As shown in Supplementary Fig. 4, glycine receptor, GluA1 and GluA2 subunits of AMPA receptor co-localize with calbindin, a marker of Purkinje cells. It seems therefore likely that AMPAR are being modulated by extracellular cGMP through glycine receptors in cerebellum located in Purkinje cells, probably in parallel fiber-Purkinje cell synapse.

Parallel fiber activity generates fast post-synaptic currents via AMPAR resulting in Purkinje cell depolarization. AMPAR in the parallel fiber-Purkinje cell synapse play a major role in the modulation of synaptic plasticity in cerebellum, both of LTP and, especially, of LTD, which have been proposed as mechanisms modulating motor learning^41–43^. LTD is induced by promoting internalization of AMPA receptors, which is mediated by phosphorylation by PKC^41,42^. It is therefore likely that extracellular cGMP could also modulate or affect the formation of LTD and, subsequently of motor learning.

We show that extracellular cGMP increases membrane expression of GluA1 and reduces that of GluA2. This would result in a change in the proportion of GluA1/GluA2 subunits of AMPA receptors present in the membrane, therefore, altering their functional properties^2–4^. GluA2-lacking AMPA receptors are Ca^2+^ permeable, blocked by polyamines and exhibit large single-channel currents. In contrast, GluA2-containing receptors are Ca^2+^ impermeable, lack polyamine sensitivity and exhibit small amplitude unitary events^5,44^. The reduced proportion of GluA2/GluA1 subunits induced by extracellular cGMP would result in altered glutamatergic neurotransmission. For similar pre-synaptic release of glutamate in the synapsis, the increase in Ca^2+^ in the post-synaptic neurons through AMPA receptors would be enhanced by extracellular cGMP. The larger increase in Ca^2+^ would activate different signal transduction pathways leading to different biological effects which may include modulation of learning in the Y maze or spatial learning and memory in the radial maze^24,28^.

Extracellular cGMP is released from most cell types through ATP-dependent transporters^45^. It increases as a consequence of glutamate receptors activation both in primary cultures of cerebellar neurons^27^ and in the extracellular fluid in rat cerebellum *in vivo*, as shown by microdialysis. In fact, measuring extracellular cGMP *in vivo* in freely moving rats by microdialysis has been used as a procedure to assess NMDA receptor activation *in vivo* ^46–48^. Extracellular cGMP levels are modulated by multiple mechanisms, including the activity of different types of glutamate (NMDA, AMPA and metabotropic) or GABA receptors^46,47,49–52^ . Extracellular cGMP in cerebellum could therefore be considered as a “sensor” of synaptic activity. The modulation by extracellular cGMP of membrane expression of GluA1 and GluA2 subunits of AMPA receptors could serve to adapt activation of these receptors to the synaptic activity of surrounding cells.

It is well known that intracellular cGMP modulates some forms of learning and memory.

Sildenafil or zaprinast, inhibitors of phosphodiesterase-5 which increase intracellular cGMP levels, modulate the ability to learn a conditional discrimination task in the Y-maze^53^ and spatial learning assessed in the radial and Morris Water Mazes^54^.

It has been recently shown that administration of extracellular cGMP *in vivo* also modulates some aspects of spatial memory, restoring reference memory but not working memory in rats with chronic hyperammonemia^24^. This suggests that extracellular cGMP also modulates some hippocampus-dependent forms of learning.

Extracellular cGMP in cerebellum also modulates the ability to learn a Y-maze task in rats *in vivo*. Rats with chronic hyperammonemia show reduced extracellular cGMP levels in cerebellum and impaired ability to learn this task. Chronic intracerebral administration of extracellular cGMP normalized extracellular cGMP in cerebellum of hyperammonemic rats and restored their ability to learn the Y maze task^28^ . The ability to learn the Y maze task depends on the extracellular cGMP concentration in cerebellum. There is an optimal range of extracellular cGMP for which learning of this task is optimal while too high or too low levels of extracellular cGMP result in reduced learning ability^28^.

Extracellular cGMP in cerebellum changes with age and in pathological conditions. It is reduced with ageing and is much lower in 7-months-old than in 3-months old rats^55^. Extracellular cGMP is also reduced in cerebellum of rats with chronic hyperammonemia or hepatic encephalopathy^53^. Changes in extracellular cGMP associated to ageing or pathological situations would alter the subunit composition of AMPA receptors in the membrane and, therefore, associated neurotransmission and cognitive function.

There are a number of pathological situations in which membrane expression of the AMPA receptor subunits is altered, resulting in altered glutamatergic neurotransmission. This occurs for example in Rett syndrome^56^, schizophrenia^57^, hyperammonemia^24,58^; hepatic encephalopathy^54,59^; chronic stress^60^, inflammation^61^, by beta-amyloid^62,63^ or with ageing^64^. The steps identified in the present work may be therapeutic targets to improve neurotransmission and neurological function in these pathological situations.

In summary, this work unveils a new physiological role of extracellular cGMP in cerebellum, which modulates membrane expression of GluA1 and GluA2 subunits of AMPA receptor *in vivo*. The study also sheds light on several important components of the intracellular response to extracellular cGMP. Extracellular cGMP reduces glycine receptor activation, modulating intracellular calcium^28^, cAMP, protein kinases and phosphatases, and GluA1 and GluA2 phosphorylation leading to increased GluA1 and reduced GluA2 membrane expression. As the subunit composition determines functional properties of AMPAR^4,5^, the results reported support the hypothesis that extracellular cGMP modulates AMPAR functional properties and glutamatergic neurotransmission. The steps identified may therefore provide therapeutic targets to improve neurotransmission and neurological function in pathological situations with abnormal glutamatergic neurotransmission such as Rett syndrome, schizophrenia, chronic stress, inflammation, hyperammonemias or hepatic encephalopathy.

## Methods

### Animals

Male Wistar rats were used (220–280 g, Charles River Laboratories, Barcelona, Spain). The experiments were approved by the Príncipe Felipe Research Center and carried out in accordance with the European Communities Council Directive (86/609/EEC).

### Continuous intracerebral administration of cGMP to rats using osmotic pumps

Rats were divided in two groups. For one group of rats the osmotic pumps were filled with 240 µM cGMP (Sigma-Aldrich, Saint Louis, USA) in sterile saline. For the other group pumps were filled with the vehicle solution, sterile saline. The pumps (mini-osmotic pump, model 2004, ALZET, Cupertino, California, U.S.) were implanted in the back of the rats. These pumps released 0.25 µl per hour during 28 days and were connected to a cannula (Brain infusion kit 2, 3–5 mm, ALZET) implanted in the cerebral ventricle as in Erceg *et al*.^65^.

### Analysis of surface expression of receptors by cross-linking with BS3

Membrane surface expression was performed using the BS3 cross-linker procedure as described in Cabrera-Pastor *et al*.^66^. The BS3 technique has been used by many groups for more than 30 years to assess membrane expression of many different types of proteins. Crosslinking with BS3 was already used by Kotite *et al*.^67^ in 1984 to analyze the interaction of specific platelet membrane proteins with collagen. This BS3 procedure was already used and validated to analyze membrane expression of AMPA receptors in 1997 by Hall and Soderling^68^, who calculated surface expression of AMPA receptors as the difference in band intensities of samples incubated or not with BS3. Moreover, they compared the results obtained with the BS3 procedure with other methods such as biotinylation of surface proteins or proteolysis of surface proteins with chymotrypsin. The BS3, biotin and chymotrypsin procedures yielded essentially the same results, thus confirming the validity of the BS3 procedure to quantify membrane expression of AMPA receptors. The method has been therefore rigorously validated 20 years ago for quantification of membrane expression of AMPA receptors. After this validation, this technique has been applied to analyze membrane expression of many proteins, including AMPA receptors^24,54,58,67–73^.

Cerebellum was dissected and transverse slices (400 µm) were obtained using a vibratome, transferred to incubation wells and incubated for 30 min at 35.5 °C in Krebs buffer (in mmol/L): NaCl 119, KCl 2.5, KH_2_PO_4_ 1, NaHCO_3_ 26.2, CaCl_2_ 2.5, and glucose 11, aerated with 95% O_2_ and 5% CO_2_ at pH 7.4. cGMP (40 nM, as in Cabrera-Pastor *et al*.^28^) or the antagonist of glycine receptors strychnine (75 μM, as in Cabrera-Pastor *et al*.^28^) from Sigma were added and incubated for 20 min. The inhibitors of protein kinase C (1 µM Bisindolylmaleimide II) from Tocris; of protein kinase A (1 µM 14–22 Amide) or of CaMKII (10 µM KN62) from Calbiochem and of protein kinase G (10 µM Rp-8-pCPT-cGMPS) from Biolog were added 10 min before addition of cGMP. Inhibitors concentration was 100-fold the IC50 values. Slices were added to tubes containing ice-cold standard buffer with or without 2 mM BS3 (bis(sulfosuccinimidyl) suberate, Pierce, Rockford, IL) and incubated for 30 min at 4 °C. Cross-linking was terminated by adding 100 mM glycine (10 min, 4 °C). The slices were homogenized by sonicating for 20 s. Protein was determined by the bicinchoninic acid method (Protein Assay Reagent, Pierce, Rockford, IL, USA). Samples treated or not with BS3 were analyzed by western blot as described below. 150 µg of total protein were loaded for each sample. The surface expression of each subunit (GluA1, GluA2 and NR2A) was calculated as the difference between the intensity of the bands without BS3 (total protein) and with BS3 (non-membrane protein) as described by Hall and Soderling^68^.

### Analysis of surface expression of receptors by biotynilation

Membrane surface expression was performed using the biotin procedure as described in Williams *et al*.^74^ and Chiu *et al*.^75^ After cGMP treatment, cerebellar slices were individually biotinylated with 1 ml of sulfo-nhs-ss-biotin (“biotin”) (Pierce) at 2 mg/ml in ice-cold krebs for 1 hr at 4 °C with gentle shaking. The biotinylation solution was removed by three washes in Krebs plus 100 mM glycine and quenched in this solution by incubating at 4 °C for 45 min with gentle shaking. Biotinylated cerebellar slices were individually sonicated in lysis buffer. A portion of total lysate was reserved. The lysates were centrifuged at 20,000 × *g* at 4 °C for 60 min. The supernatant fractions were incubated with an equal volume of Immunopure immobilized monomeric neutravidin beads (Pierce) for 60 min at 4 °C. Neutravidin beads and bound biotinylated proteins were isolated by centrifugation and washed repeatedly with lysis buffer. A portion of the post-neutravidin supernatant lysate was analyzed as intracellular fraction. Biotinylated proteins were eluted from neutravidin beads by incubation at 100 °C in sample buffer containing 0.1 M dithiothreitol (DTT) to produce the eluant fraction corresponding to surface proteins. The surface expression of each subunit (GluA1 and GluA2) was analyzed by western blot.

### Analysis of protein content in cerebellum by Western blot

Homogenates of cerebellar slices were subjected to immunoblotting as in Felipo *et al*.^76^ Protein concentration in the homogenates was quantified using the bicinchoninic acid method (Protein Assay Reagent, Pierce, Rockford, IL, USA) and 30 µg of total protein was loaded per lane. Primary antibodies were against GluA1, GluA2 (1:250 and 1:1000 dilution, respectively), NR2A (1:1000 dilution) and GluA1 phosphorylated at Ser831 (1:1000 dilution) from Merck Millipore (Darmstadt, Germany); GluA1 phosphorylated at Ser845, GluA2 phosphorylated at Ser880, CaMKII phosphorylated at Thr286 and phosphatase 1(1:1000 dilution) from Abcam (Cambridge, MA); CaMKII (1:1000 dilution) from Thermo Fisher Scientific (Waltham, MA USA). Protein phosphatase inhibitor-1 phosphorylated at Thr35 (1:500 dilution) from Santa Cruz Biotechnology, Inc (Heidelberg, Germany). As a control for protein loading, the same membranes used to quantify the amount of proteins (e.g. GluA1, GluA2) were incubated with an antibody against Actin (1:5000) from Abcam (Cambridge, MA). Secondary antibodies were anti-rabbit, anti-goat or anti-mouse IgG, 1:2000 dilution conjugated with alkaline phosphatase from Sigma (St. Louis, MO). The images were captured using the ScanJet 5300C (Hewlett-Packard, Amsterdam, Netherlands) and band intensities quantified using the Alpha Imager 2200, version 3.1.2 (AlphaInnotech Corporation, San Francisco). Phosphorylation levels at Ser 831 and Ser 845, Ser880 and Thr286 were normalized respect to total protein levels of GluA1, GluA2 and CaMKII, respectively.

### Determination of cAMP in cerebellar slices

Cyclic AMP was determined with cAMP immunoassay kit from abcam. Slices were incubated with or without cGMP (40 nM) or strychnine (75 µM) during 20 min and immediately homogenized in the kit assay buffer. Samples were boiled at 100 °C for 5 min and centrifuged (14000 g, 5 min). cAMP was measured in the supernatant diluted 1:50. Pellets were suspended in 0.5 M NaOH and protein concentration was measured using the Lowry’s procedure.

### Statistical analysis

Results are expressed as mean ± standard error. All statistical analyses were performed using the software program GraphPad Prism. Normality was assessed using the D’Agostino and Pearson Omnibus test and the Shapiro-Wilk normality tests. Differences in variances of normally distributed data were assessed using Bartlett’s test. Data with the same variance across groups were analyzed by one-way analysis of variance (ANOVA) followed by Bonferroni’s post-hoc test. Data with different variance across groups were analyzed using the non-parametric Kruskal-Wallis test followed by Dunnett’s post-hoc test. When only two groups were compared the unpaired Student’s t-test (for normally distributed data) was used. A confidence level of 95% was accepted as significant. The statistical procedure used in each case is indicated in the corresponding Figure legend.

## Electronic supplementary material

Supplementary information

## References

[1] Keinänen K (1990). family of AMPA-selective glutamate receptors. Science..

[2] Shi S, Hayashi Y, Esteban JA, Malinow R (2001). Subunit-specific rules governing AMPA receptor trafficking to synapses in hippocampal pyramidal neurons. Cell..

[3] Bredt DS, Nicoll RA (2003). AMPA receptor trafficking at excitatory synapses. Neuron.

[4] Huganir RL, Nicoll RA (2013). AMPARs and synaptic plasticity: the last 25 years. Neuron.

[5] Hollmann M, Hartley M, Heinemann S (1991). Ca^2+^ permeability of KA-AMPA-gated glutamate receptor channels depends on subunit composition. Science.

[6] Llano I, Marty A, Armstrong CM, Konnerth A (1991). Synaptic and agonist-induced excitatory currents of Purkinje neurons in rat cerebellar slices. J. Physiol. (London).

[7] Tempia F (1996). Fractional calcium current through neuronal AMPA-receptor channels with a low calcium permeability. J. Neurosci..

[8] Ho VM, Lee JA, Martin KC (2011). The cell biology of synaptic plasticity. Science.

[9] Wang, G., Gilbert, J. & Man, H.Y. AMPA receptor trafficking in homeostatic synaptic plasticity: functional molecules and signaling cascades. *Neural Plast*. 825364 (2012). 10.1155/2012/825364 PMC3359728 22655210

[10] Chater TE, Goda Y (2014). The role of AMPA receptors in postsynaptic mechanisms of synaptic plasticity. Front. Cell Neurosci..

[11] Chung HJ, Xia J, Scannevin RH, Zhang X, Huganir RL (2000). Phosphorylation of the AMPA receptor subunit GluR2 differentially regulates its interaction with PDZ domain-containing proteins. J. Neurosci..

[12] Perez JL (2001). PICK1 targets activated protein kinase C alpha to AMPA receptor clusters in spines of hippocampal neurons and reduces surface levels of the AMPA-type glutamate receptor subunit 2. J. Neurosci..

[13] Chung HJ, Steinberg JP, Huganir RL, Linden DJ (2003). Requirement of AMPA receptor GluR2 phosphorylation for cerebellar long-term depression. Science.

[14] Terashima A (2004). Regulation of synaptic strength and AMPA receptor subunit composition by PICK1. J. Neurosci..

[15] Barria A, Derkach V, Soderling T (1997). Identification of the Ca2+/calmodulin-dependent protein kinase II regulatory phosphorylation site in the alpha-amino-3-hydroxyl-5-methyl-4-isoxazole-propionate-type glutamate receptor. J. Biol. Chem..

[16] Mammen AL, Kameyama K, Roche KW, Huganir RL (1997). Phosphorylation of the alpha-amino-3-hydroxy-5-methylisoxazole4-propionic acid receptor GluR1 subunit by calcium/calmodulin-dependent kinase II. J. Biol. Chem..

[17] Benke TA, Lüthi A, Isaac JT, Collingridge GL (1998). Modulation of AMPA receptor unitary conductance by synaptic activity. Nature..

[18] Esteban JA (2003). PKA phosphorylation of AMPA receptor subunits controls synaptic trafficking underlying plasticity. Nat. Neurosci..

[19] Blitzer RD (1998). Gating of CaMKII by cAMP-regulated protein phosphatase activity during LTP. Science.

[20] Jin XH, Siragy HM, Carey RM (2001). Renal interstitial cGMP mediates natriuresis by direct tubule mechanism. Hypertension.

[21] Zimmerman NP, Brownfield MS, DeVente J, Bass P, Oaks JA (2008). cGMP secreted from the tapeworm Hymenolepis diminuta is a signal molecule to the host intestine. J. Parasitol..

[22] Touyz RM, Picard S, Schiffrin EL, Deschepper CF (1997). Cyclic GMP inhibits a pharmacologically distinct Na^+^/H^+^ exchanger variant in cultured rat astrocytes via an extracellular site of action. J. Neurochem..

[23] Bukanova JV, Solntseva EI, Kondratenko RV, Skrebitsky VG (2014). Glycine receptor in hippocampal neurons as a target for action of extracellular cyclic nucleotides. Neurosci. Lett..

[24] Cabrera-Pastor A (2016). *In vivo* administration of extracellular cGMP normalizes TNF-α and membrane expression of AMPA receptors in hippocampus and spatial reference memory but not IL-1β, NMDA receptors in membrane and working memory in hyperammonemic rats. Brain Behav. Immun..

[25] Linden DJ, Dawson TM, Dawson VL (1995). An evaluation of the nitric oxide/cGMP/cGMP-dependent protein kinase cascade in the induction of cerebellar long-term depression in culture. J. Neurosci..

[26] Cervetto C, Maura G, Marcoli M (2010). Inhibition of presynaptic release-facilitatory kainate autoreceptors by extracellular cyclic GMP. J. Pharmacol. Exp. Ther..

[27] Montoliu C, Llansola M, Kosenko E, Corbalan R, Felipo V (1999). Role of cyclic GMP in glutamate neurotoxicity in primary cultures of cerebellar neurons. Neuropharmacology.

[28] Cabrera-Pastor A, Malaguarnera M, Taoro-Gonzalez L, Llansola M, Felipo V (2016). Extracellular cGMP Modulates Learning Biphasically by Modulating Glycine Receptors, CaMKII and Glutamate-Nitric Oxide-cGMP Pathway. Sci. Rep..

[29] Wayman GA, Lee YS, Tokumitsu H, Silva AJ, Soderling TR (2008). Calmodulin-kinases: modulators of neuronal development and plasticity. Neuron..

[30] Lou LL, Schulman H (1989). Distinct autophosphorylation sites sequentially produce autonomy and inhibition of the multifunctional Ca2þ/calmodulin-dependent protein kinase. J. Neurosci..

[31] Hashimoto Y, Schworer CM, Colbran RJ, Soderling TR (1987). Autophosphorylation of Ca2+/calmodulin-dependent protein kinase II. Effects on total and Ca2þ-independent activities and kinetic parameters. J. Biol. Chem..

[32] Kitagawa Y, Hirano T, Kawaguchi SY (2009). Prediction and validation of a mechanism to control the threshold for inhibitory synaptic plasticity. Mol. Syst. Biol..

[33] Colledge M (2000). Targeting of PKA to glutamate receptors through a MAGUK-AKAP complex. Neuron..

[34] Ostroveanu A (2007). A-kinase anchoring protein 150 in the mouse brain is concentrated in areas involved in learning and memory. Brain Res..

[35] Vaello ML, Ruiz-Gómez A, Lerma J, Mayor F (1994). Modulation of inhibitory glycine receptors by phosphorylation by protein kinase C and cAMP-dependent protein kinase. Biol. Chem..

[36] Ren J, Ye JH, McArdle JJ (1998). cAMP-dependent protein kinase modulation of glycine-activated chloride current in neurons freshly isolated from rat ventral tegmental area. Brain Res..

[37] Velázquez-Flores MÁ, Salceda R (2011). Glycine receptor internalization by protein kinases activation. Synapse..

[38] Kawaguchi S, Hirano T (2000). Suppression of inhibitory synaptic potentiation by presynaptic activity through postsynaptic GABA(B) receptors in a Purkinje neuron. Neuron.

[39] Hirano T, Kawaguchi SY (2014). Regulation and functional roles of rebound potentiation at cerebellar stellate cell-Purkinje cell synapses. Front. Cell Neurosci..

[40] Masugi-Tokita M (2007). Number and density of AMPA receptors in individual synapses in the rat cerebellum as revealed by SDS-digested freeze-fracture replica labeling. J. Neurosci..

[41] Hirano T (2013). Long-term depression and other synaptic plasticity in the cerebellum. Proc. Jpn. Acad. Ser. B. Phys. Biol. Sci..

[42] Hoxha, E., Tempia, F., Lippiello, P. & Miniaci, M. C. Modulation, Plasticity and Pathophysiology of the Parallel Fiber-Purkinje Cell Synapse. *Front. Synaptic Neurosci*. **8**:35. eCollection (2016). 10.3389/fnsyn.2016.00035 PMC5093118 27857688

[43] Freeman JH (2015). Cerebellar learning mechanisms. Brain Res..

[44] Washburn MS, Dingledine R (1996). Block of alpha-amino-3-hydroxy-5-methyl-4-isoxazolepropionic acid (AMPA) receptors by polyamines and polyamine toxins. J. Pharmacol. Exp. Ther..

[45] Sager G (2004). Cyclic GMP transporters. Neurochem. Int..

[46] Hermenegildo C (1998). Chronic hyperammonemia impairs the glutamate-nitric oxide-cyclic GMP pathway in cerebellar neurons in culture and in the rat *in vivo*. Eur. J. Neurosci..

[47] Hermenegildo C, Monfort P, Felipo V (2000). Activation of N-methyl-D-aspartate receptors in rat brain *in vivo* following acute ammonia intoxication: characterization by *in vivo* brain microdialysis. Hepatology.

[48] Fedele E, Raiteri M (1999). *In vivo* studies of the cerebral glutamate receptor/NO/cGMP pathway. Prog. Neurobiol..

[49] Fedele E, Bisaglia M, Raiteri M (1997). D-serine modulates the NMDA receptor/nitric oxide/cGMP pathway in the rat cerebellum during *in vivo* microdialysis. Naunyn Schmiedebergs Arch. Pharmacol..

[50] Fedele E, Ansaldo MA, Varnier G, Raiteri M (2000). Benzodiazepine-sensitive GABA(A) receptors limit the activity of the NMDA/NO/cyclic GMP pathway: a microdialysis study in the cerebellum of freely moving rats. J. Neurochem..

[51] Cauli O, Mansouri MT, Agusti A, Felipo V (2009). Hyperammonemia increases GABAergic tone in the cerebellum but decreases it in the rat cortex. Gastroenterology..

[52] Boix J, Llansola M, Cabrera-Pastor A, Felipo V (2011). Metabotropic glutamate receptor 5 modulates the nitric oxide-cGMP pathway in cerebellum *in vivo* through activation of AMPA receptors. Neurochem. Int..

[53] Erceg S (2005). Oral administration of sildenafil restores learning ability in rats with hyperammonemia and with portacaval shunts. Hepatology.

[54] Hernández-Rabaza V (2015). Sildenafil reduces neuroinflammation and restores spatial learning in rats with hepatic encephalopathy: underlying mechanisms. J. Neuroinflammation.

[55] Piedrafita B, Cauli O, Montoliu C, Felipo V (2007). The function of the glutamate-nitric oxide-cGMP pathway in brain *in vivo* and learning ability decrease in parallel in mature compared with young rats. Learn. Mem..

[56] Li W, Xu X, Pozzo-Miller L (2016). Excitatory synapses are stronger in the hippocampus of Rett syndrome mice due to altered synaptic trafficking of AMPA-type glutamate receptors. Proc. Natl. Acad. Sci. USA.

[57] Tucholski J (2013). Abnormal N-linked glycosylation of cortical AMPA receptor subunits in schizophrenia. Schizophr. Res..

[58] Hernández-Rabaza V (2016). Hyperammonemia induces glial activation, neuroinflammation and alters neurotransmitter receptors in hippocampus, impairing spatial learning: reversal by sulforaphane. J. Neuroinflammation.

[59] Dadsetan S (2016). Reducing Peripheral Inflammation with Infliximab Reduces Neuroinflammation and Improves Cognition in Rats with Hepatic Encephalopathy. Front. Mol. Neurosci..

[60] Kallarackal AJ (2013). Chronic stress induces a selective decrease in AMPA receptor-mediated synaptic excitation at hippocampal temporoammonic-CA1 synapses. J. Neurosci..

[61] Kopach O (2011). Inflammation alters trafficking of extrasynaptic AMPA receptors in tonically firing lamina II neurons of the rat spinal dorsal horn. Pain.

[62] Monfort P, Felipo V (2010). Amyloid-β impairs, and ibuprofen restores, the cGMP pathway, synaptic expression of AMPA receptors and long-term potentiation in the hippocampus. J. Alzheimers Dis..

[63] Gilbert J (2016). β-Amyloid triggers aberrant over-scaling of homeostatic synaptic plasticity. Acta Neuropathol. Commun..

[64] Chen Z, Stockwell J, Cayabyab FS (2016). Adenosine A1 Receptor-Mediated Endocytosis of AMPA Receptors Contributes to Impairments in Long-Term Potentiation (LTP) in the Middle-Aged Rat Hippocampus. Neurochem. Res..

[65] Erceg S (2005). Restoration of learning ability in hyperammonemic rats by increasing extracellular cGMP in brain. Brain Res..

[66] Cabrera-Pastor A, Taoro L, Llansola M, Felipo V (2015). Roles of the NMDA receptor and EAAC1 transporter in the modulation of extracellular glutamate by low and high affinity AMPA receptors in the cerebellum *in vivo*: differential alteration in chronic hyperammonemia. ACS Chem. Neurosci..

[67] Kotite NJ, Staros JV, Cunningham LW (1984). Interaction of specific platelet membrane proteins with collagen: evidence from chemical cross-linking. Biochemistry.

[68] Hall RA, Soderling TR (1997). Quantitation of AMPA receptor surface expression in cultured hippocampal neurons. Neuroscience..

[69] Hall RA, Soderling TR (1997). Differential surface expression and phosphorylation of the N-methyl-D-aspartate receptor subunits NR1 and NR2 in cultured hippocampal neurons. J. Biol. Chem..

[70] Hall RA, Hansen A, Andersen PH, Soderling TR (1997). Surface expression of the AMPA receptor subunits GluR1, GluR2, and GluR4 in stably transfected baby hamster kidney cells. J. Neurochem..

[71] Archibald K, Perry MJ, Molnár E, Henley JM (1998). Surface expression and metabolic half-life of AMPA receptors in cultured rat cerebellar granule cells. Neuropharmacology.

[72] Boudreau AC, Wolf ME (2005). Behavioral sensitization to cocaine is associated with increased AMPA receptor surface expression in the nucleus accumbens. J. Neurosci..

[73] Gould TD (2008). Involvement of AMPA receptors in the antidepressant-like effects of lithium in the mouse tail suspension test and forced swim test. Neuropharmacology.

[74] Williams R, Fuchs JR, Green JT, Morielli A (2012). Cellular mechanisms and behavioral consequences of Kv1.2. Regulation in the rat cerebellum. J. Neurosci..

[75] Chiu CS (2002). Number, density, and Surface/cytoplasmic distribution of GABA transporters at presynaptic structures of knock in mice carrying GABA transporter subtype 1-Green fluorescent protein fusions. J. Neurosci..

[76] Felipo V, Minana MD, Azorin I, Grisolia S (1988). Induction of rat brain tubulin following ammonium ingestion. J. Neurochem..

