# Supplementary material for: Extracellular Cyclic GMP Modulates Membrane Expression of The GluA1 and GluA2 Subunits of AMPA Receptor in Cerebellum: Molecular Mechanisms Involved
Source: Sci Rep. 2017 Dec 15;7:17656. doi: 10.1038/s41598-017-18024-3 (PMC5732250; doi:10.1038/s41598-017-18024-3)
Supplement: Supplementary file 1 — Supplementary information [file 41598_2017_18024_MOESM1_ESM.pdf]

TITLE OF MANUSCRIPT:

EXTRACELLULAR CYCLIC GMP MODULATES MEMBRANE EXPRESSION OF THE GluA1 AND GluA2 SUBUNITS OF AMPA RECEPTOR IN CEREBELLUM: MOLECULAR MECHANISMS INVOLVED.

Andrea Cabrera-Pastor[1], Lucas Taoro-González[1], Amparo N. Cuñat[1], David Canet-López[1], Tiziano Balzano[1], Vicente Felipo[1]

[1]Laboratory of Neurobiology, Centro de Investigación Príncipe Felipe, 46012, Valencia, Spain

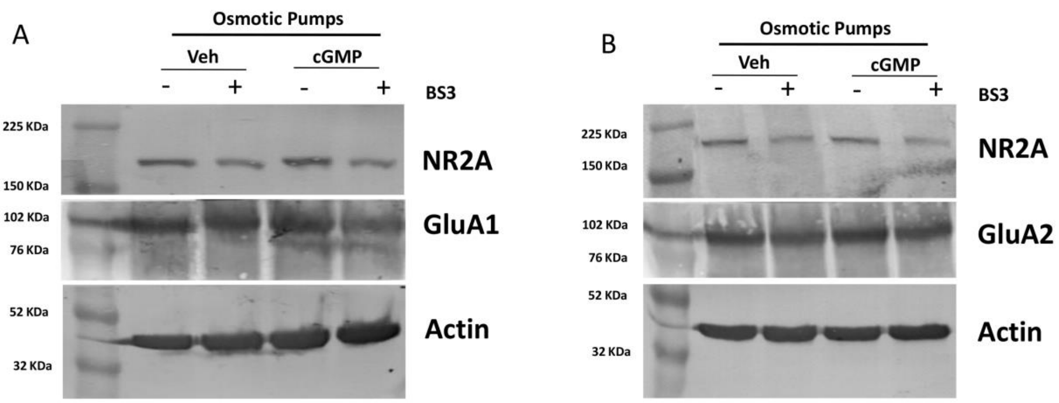

Supplementary Figure 1. Representative images of western blot membranes from the same gel, cut and incubated with antibodies against NR2A, GluA1 and actin, as a loading control (Suppl. Fig. 1A) or with NR2A, GluA2 and actin (Suppl. Fig. 1B) using BS3 crosslinking procedure as described in Methods.

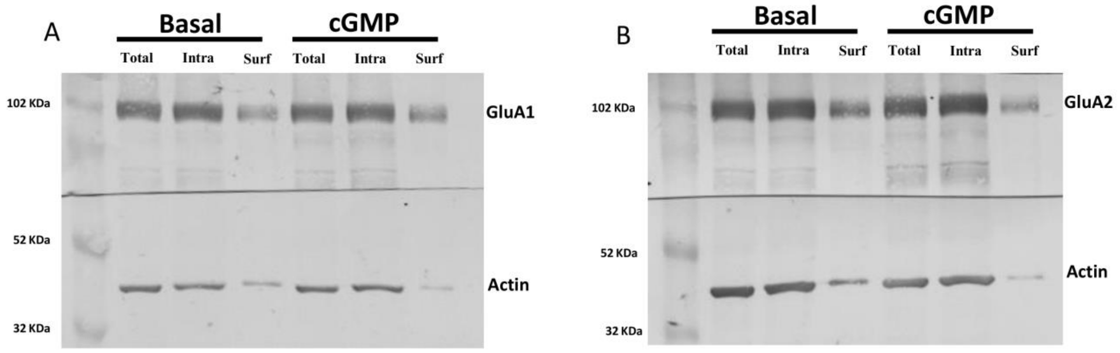

Supplementary Figure 2. Representative images of western blot membranes incubated with antibodies against GluA1 and actin, as a loading control (Suppl. Fig. 2A) or with GluA2 and actin (Suppl. Fig. 2B) using the biotinylation procedure as described in Methods. Total, intracellular and surface content of each protein are represented in absence (basal) or presence of cGMP treatment.

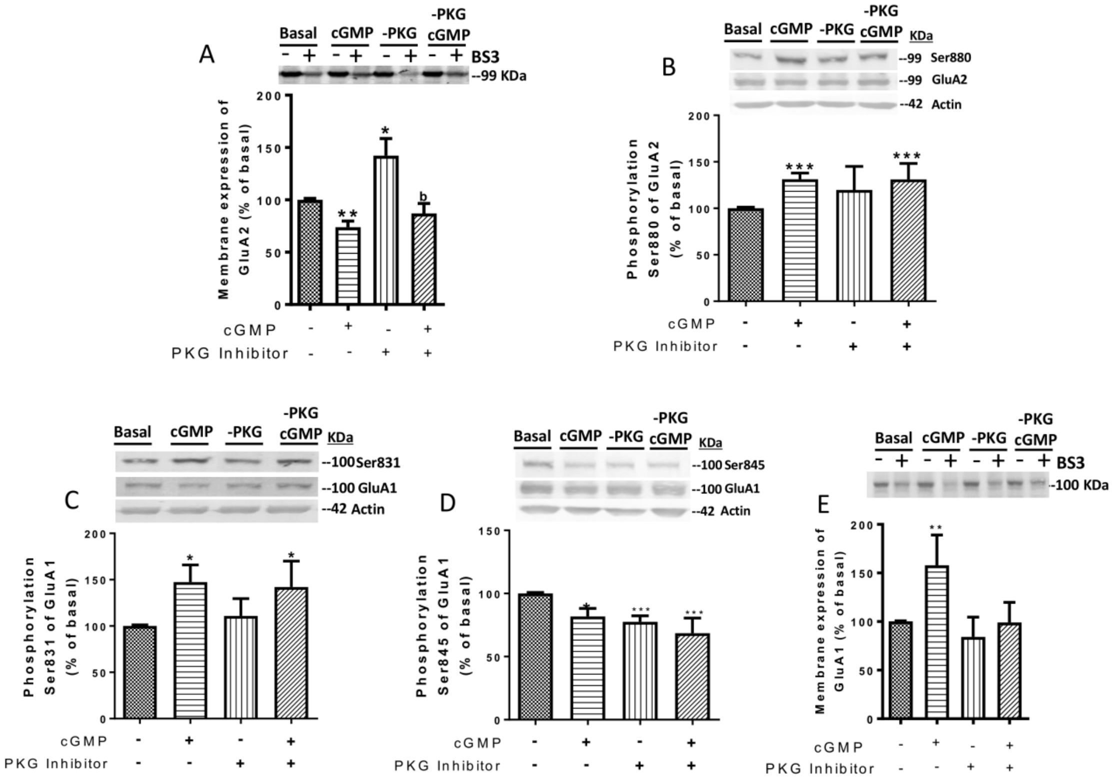

Supplementary Figure 3. Inhibition of PKG does not affect the changes in phosphorylation at Ser880 and at Ser831 (Supp. Fig. 3B and 3C) and membrane expression of GluA2 (Supp. Fig. 3A) induced by extracellular cGMP but affect membrane expression of GluA1 (Supp. Fig. 3E) and phosphorylation of GluA1 at Ser845 (Supp. Fig. 3D). Extracellular cGMP was added to cerebellar slices in the absence or the presence of an inhibitor of PKG (10 µM Rp-8-pCPT-cGMPS) and membrane expression of GluA2 (A) and GluA1 (E) and its phosphorylation at Ser880 (B), at Ser831 (C) and at Ser845 (D), respectively, were analyzed as described in Methods. Data were analyzed by one-way analysis of variance (in D) and by the non-parametric Kruskal-Wallis test (in A-C, E). Values are expressed as percentage of basal and are the mean±SEM of 8-12 rats. Values significantly different from basal are indicated by asterisk *$p<0.05$, **$p<0.01$, ***$p<0.001$. Values significantly different from PKG inhibitor treatment are indicated by b $p<0.05$.

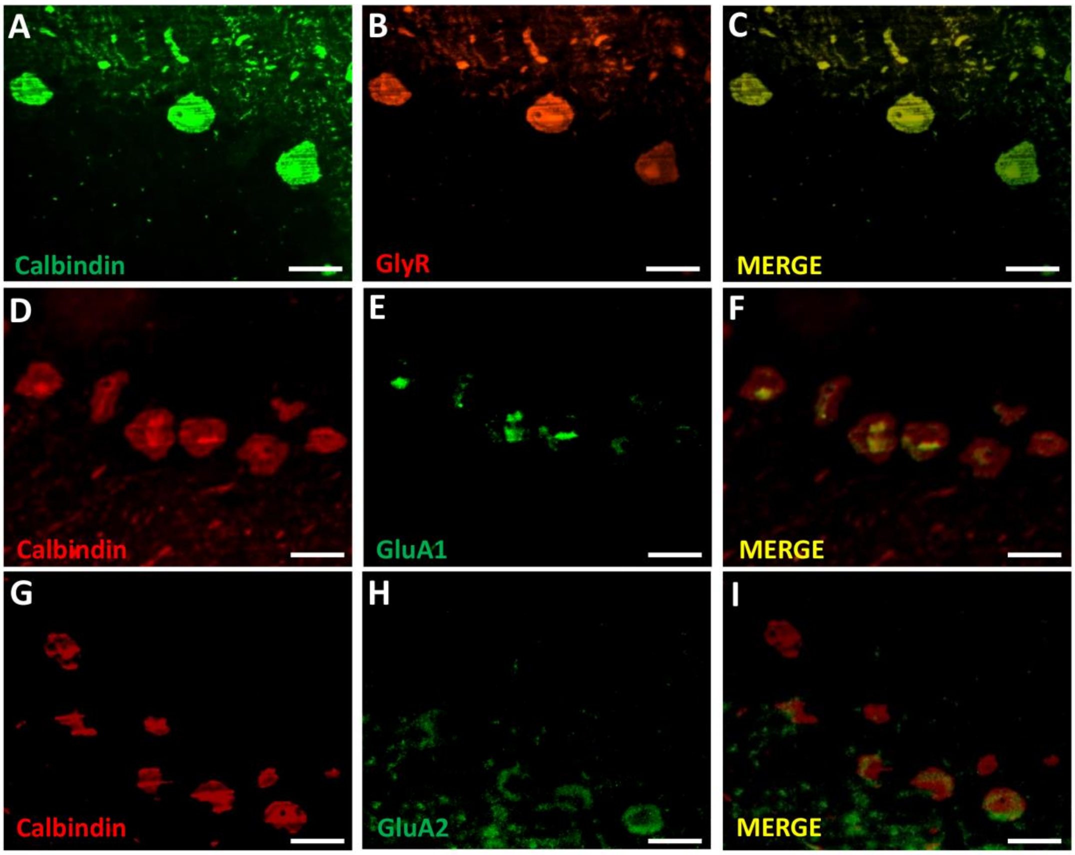

Supplementary Figure 4. Glycine receptors, GluA1 and GluA2 subunits of AMPA receptors co-localize with calbindin, a marker of Purkinje cells. Immunofluorescence was performed in cerebellum of rats as described in Supplementary Methods. Double fluorescence staining of the Purkinje cells marker (A, D, G), Glycine receptor (B), GluA1 (E) and GluA2 (H) subunits and merge (C, F, I) showing co-localization (Scale bar= 20 µm).

Supplementary Methods

Brain immunofluorescence. Rats were anaesthetized with sodium pentobarbital and transcardially perfused with 0.9 % saline followed by 4 % paraformaldehyde in 0.1 M phosphate buffer (pH 7.4). Brains were removed and post-fixed in the same fixative solution for 24 h at 4 °C. Free-floating sections of cerebellum (30 µm) were cut using vibratome. Sections were washed in 0.1 M phosphate buffer and blocked with normal serum from the same species as the secondary antibody before being incubated overnight with primary antibodies (Glycine receptor 1:500; GLUA1, 1:200; GLUA2,

1:200; Calbindin, 1:1000), diluted in blocking buffer and secondary fluorescent antibodies (Alexa fluor 488 and Alexa fluor 647, 1:400). The nuclei were stained with DAPI and sections were mounted on slides and coverslipped. The images were observed under confocal microscope (Leica TCS-SP2-AOBS) and photographically recorded (63x).
